# Involvement of C-terminal truncation mutation of kinesin-5 in resistance to kinesin-5 inhibitor

**DOI:** 10.1371/journal.pone.0209296

**Published:** 2018-12-17

**Authors:** Eri Saeki, Shinji Yasuhira, Masahiko Shibazaki, Hiroshi Tada, Minoru Doita, Tomoyuki Masuda, Chihaya Maesawa

**Affiliations:** 1 Department of Tumor Biology, Institute of Biomedical Sciences, Iwate Medical University, 2-1-1 Nishitokuta, Yahaba-cho, Shiwa-gun, Iwate, Japan; 2 Department of Orthopaedic Surgery, School of Medicine, Iwate Medical University, 16-1 Uchimaru, Morioka-shi, Iwate, Japan; 3 Department of Pathology, School of Medicine, Iwate Medical University, 2-1-1 Nishitokuta, Yahaba-cho, Shiwa-gun, Iwate, Japan; Taipei Medical University College of Medicine, TAIWAN

## Abstract

Cultured cells easily develop resistance to kinesin-5 inhibitors (K5Is) often by overexpressing a related motor protein, kinesin-12/KIF15, or by acquiring mutations in the N-terminal motor domain of kinesin-5/KIF11 itself. We aimed to identify novel mechanisms responsible for resistance to S-trityl L-cysteine (STLC), one of the K5Is, using human osteosarcoma cell lines. Among six lines examined, U-2OS and HOS survived chronic STLC treatment and gave rise to resistant cells with IC_50_s at least 10-fold higher than those of the respective parental lines. Depletion of KIF15 largely eliminated the acquired K5I resistance in both cases, consistent with the proposed notion that KIF15 is indispensable for it. In contrast to the KIF11-independent property of the cells derived from HOS, those derived from U-2OS still required KIF11 for their growth and, intriguingly, expressed a C-terminal truncated variant of KIF11 resulting from a frame shift mutation (S1017fs). All of the isolated clones harbored the same mutation, suggesting its clonal expansion in the cell population due to the growth advantage during chronic STLC treatment. Transgenic expression of KIF11^S1017fs^ in the parental U-2OS cells, as well as in HeLa cells, conferred a moderate but reproducible STLC resistance, probably owing to STLC-resistant localization of the mutant KIF11 on mitotic spindle. Our observations indicate that both KIF15 and the C-terminal-truncated KIF11 contributes to the STLC resistance of the U-2OS derived cells.

## Introduction

Bipolar spindle assembly is a prerequisite for faithful segregation of duplicated chromatids into a pair of daughter cells in mitosis. While maintenance of the assembled spindle in metaphase is accomplished by a set of cellular proteins with a certain degree of redundancy, centrosome separation beginning in prophase and bipolarity establishment in prometaphase may be carried out predominantly by a single class of essential plus-end-directed motor protein, kinesin-5, in most eukaryotes [1,2]. This makes KIF11/Eg5, a human kinesin-5 ortholog, an attractive target for cancer chemotherapeutics, and several chemicals that interfere with its activity (kinesin-5 inhibitors or K5Is), and hopefully would impede proliferation of tumor cells [3–5], have been developed. Subsequent studies, however, have revealed that the bipolarity establishment step also has cryptic redundancy, as cultured cells often develop resistance to K5Is by activating one of the alternative pathways [6–8]. The establishment of bipolarity requires two distinct activities of KIF11 on interpolar microtubules (MTs), i.e. their bundling and sliding. A typical mechanism of acquired K5I-resistance involves elevated expression of another plus-end-directed motor, kinesin-12/KIF15. In unperturbed mitosis, KIF15 resides exclusively on kinetochore-MTs and plays a minor role in maintenance of bipolarity. When overexpressed, part of KIF15 somehow mislocalizes to interpolar MTs and takes over both the bundling and sliding roles from KIF11 in K5I-perturbed mitosis to establish bipolarity [7]. In this situation, however, it is not very clear how an increased KIF15 level would accompany redistribution to interpolar MTs. Another recently reported mechanism for the resistance involves a “rigor” mutant of KIF11 that acquires higher affinity for MTs in exchange for loss of sliding activity [8]. Increased affinity leads to augmented MT bundling, which may prompt the redistribution of KIF15 to interpolar MTs without the need for an increased level of expression. Here, two jobs—bundling and sliding—are divided between the mutant KIF11 and the wild-type KIF15. In either case, if bundling activity is supplied by some means, KIF15 can assemble the mitotic spindle employing its sliding activity, arguing that KIF15 may be universally required in order for cells to acquire resistance to K5Is.

Our interest in the present study was to clarify whether the requirement for KIF15 is a general feature, since most of the previous studies of acquired K5I resistance have employed only a limited number of cell lines, such as HeLa cells. We were able to obtain STLC-resistant cells from two osteosarcoma cell lines out of six examined, and show that their resistance was in fact mostly dependent on KIF15, whereas stable resistance was not acquired by the remaining four cell lines, despite KIF15 expression. Intriguingly, the STLC resistance of U-2OS-derived cells was dependent on not only KIF15 but also the expression of a novel C-terminal-truncated mutant of KIF11 to some extent. Our findings appear to indicate a previously undocumented function of the KIF11 C-terminus.

## Materials and methods

### Cell culture and media

Six osteosarcomas cell lines (U-2OS, HOS, MG-63, G-292, NY and HuO9) and HeLa cells from our laboratory stock were used for the present study. Cell-line identities were verified by STR analysis using the PowerPlex 16 System (Promega, Fitchburg, WI, USA) followed by matching with STR data in Cellosaurus of ExPASy (https://web.expasy.org/cellosaurus/). Absence of mycoplasma contamination was verified with DAPI staining regularly during the experiments. U-2OS and HOS were cultured in McCoy’s 5A medium (Thermo Fisher Scientific, Waltham, MA, USA), and MG-63, G-292, NY, HuO9 and HeLa were cultured in RPMI 1640 medium (Thermo Fisher Scientific), both supplemented with 10% fetal bovine serum in a humidified atmosphere with 5% CO2 at 37°C. For lentivirus packaging, 293T cells obtained from RIKEN BRC Cell Bank were used as described below. Selection for resistance to S-trityl L-cysteine (STLC) was done by culturing the osteosarcoma cell lines in appropriate medium containing 2 μM STLC (164739, Sigma-Aldrich, St. Louis, MO, USA) for 2 weeks, and the STLC concentration was gradually increased up to 8 μM during the following 4 weeks. Among the resistant cells derived from U-2OS, several clones were isolated by limiting dilution. STLC-resistant cells were maintained in the presence of 4 or 8 μM STLC and inoculated into medium lacking STLC immediately prior to the experiments.

### Gene knockdown

Silencer Select Validated siRNAs (s7903 for *KIF11*, s32546 or s32547 for *KIF15*, and s23499 for *BICD2*) and Silencer Select Negative Control No. 1 siRNA (4390844) were purchased from Thermo Fisher Scientific. Liposomes containing siRNA were prepared using Lipofectamine RNAiMAX Transfection Reagent (Thermo Fisher Scientific). For the experiments evaluating KIF15/BICD2-dependency of acquired STLC-resistance, two thousand cells were inoculated into each well of 96-well plates in the presence of 10 nM siRNA 24 h prior to STLC treatment. For the experiments examining KIF11/KIF15-dependency of unperturbed cell growth, the same number of the cells were inoculated and liposomes containing siRNA were added 24 h later. To assess knockdown efficiency, protein samples were prepared from the cells transfected with the siRNA for 48 h and subjected to immunoblotting analysis.

### Cell viability assay

Cell viability after the STLC treatment or *KIF11*/*KIF15* knockdown was evaluated with a water-soluble tetrazolium salts-based assay. After the treatment, the cells were incubated with fresh medium containing 10% of Cell Counting Kit-8 (Dojindo, Kumamoto, Japan) for an additional few hours, and then the absorbance at 450 nm was measured using a Multiskan Spectrum spectrophotometer (Thermo Fisher Scientific). Incubation time was adjusted so that wells with the highest absorbance attained about 1.0. The assays were repeated at least three times with independent cell inoculation and STLC dilution. For calculation of relative viability, absorbance of the wells with no cells and that for non-treated cells were normalized to 0 and 1, respectively.

### Protein preparation and immunoblotting

Cellular protein for immunoblotting was prepared as described previously [9]. Briefly, cells in a sub-confluent state were fixed with 10% trichloroacetic acid in saline for 16 h at 4°C, and lysed in 9 M urea, 2% Triton X-100 and 1% dithiothreitol. Protein concentration was measured with a BCA protein assay kit (Merck Millipore Corporation, Billerica, MA, USA) before addition of dithiothreitol. For some experiments, cells were washed and directly lysed with 1 x SDS sample buffer. Approximately 20–30 μg of protein per lane was run on 5% or 7.5% SDS polyacrylamide gels and then transferred onto polyvinylidene fluoride transfer membranes (Pall Corporation, Port Washington, NY, USA). The membranes were blocked with 5% low-fat dried milk (Morinaga Milk Industry, Tokyo, Japan) in 1 × TBS-T for 30 min at room temperature and then immuno-reacted with an appropriate primary antibody overnight at 4°C and subsequently with HRP-conjugated secondary antibody at 1:5000 dilution for 2 h at room temperature. In most experiments, KIF11 and KIF15 were simultaneously probed. Signals were visualized with ECL Prime Western Blotting Detection Reagent (GE Healthcare Life Sciences) and chemiluminescence images were taken with ChemiDoc XRS (Bio-Rad Laboratories, Hercules, CA, USA). Intensity of the signals was quantified using ImageJ/Fiji software [10] and normalized against the signal of α-tubulin. The following primary antibodies were used at the indicated dilutions: rabbit anti-KIF11 (#14404, Cell Signaling Technology, Danvers, MA, USA, or 23333-1-AP, Proteintech Group, Inc., Rosemont, IL, USA, at 1:1000), rabbit anti-KIF15 (A302-706A, Bethyl Laboratories, Inc., Montgomery, TX, USA, or 55407-1-AP, Proteintech Group, Inc., at 1:1000), rabbit anti-BICD2 (ab117818, Abcam, Cambridge, MA, USA, at 1:500), mouse anti-α-tubulin (T5168, Sigma Aldrich, at 1:1000), and rat anti-HA (11 867 423 001, Sigma-Aldrich, at 1:1000). The following secondary antibodies were used: sheep anti-mouse IgG (NA9310V, GE Healthcare Life Sciences, Chicago, IL, USA), donkey anti-rabbit IgG (NA9340V, GE Healthcare Life Sciences), and rabbit anti-rat IgG (P0450, Agilent, Santa Clara, CA, USA). According to the manufacture, epitope for anti-KIF11 does not overlap with the truncated part of KIF11 in the present study.

### cDNA synthesis for RNA quantitation and direct sequencing

Total RNA was prepared with TRIzol (Thermo Fisher Scientific) following the manufacture’s protocol, and cDNA synthesis was primed with oligo(dT) using SuperScript III First-Strand Synthesis Super Mix (Thermo Fisher Scientific). Quantitative PCR was performed for *KIF11* and *KIF15* using TaqMan Gene Expression Assays (Assay ID: Hs 00189698_m 1 and Hs 01085295_m 1, Thermo Fisher Scientific), and the amount of specific RNA was calculated by the double delta Ct method using the amount of GAPDH mRNA as a standard. Two-tailed t-tests were used for statistical evaluation. For direct sequencing, cDNA for *KIF11* and *KIF15* was PCR-amplified from total cDNA prepared as above, purified and subjected to Sanger sequencing with BigDye Terminator (Ver. 3.1, Thermo Fisher Scientific).

### Lentiviral vector-mediated gene expression

Plasmids for lentivirus-mediated gene expression, pCAG-HIVgp, pCMV-VSV-G-RSV-Rev and pCSII-EF-MCS, were obtained from Dr. H. Miyoshi at RIKEN Tsukuba Institute. The PCR-amplified cDNA fragment for wild-type human *KIF11* was cloned into pCSII-EF-MCS, and G268dV [8] or S1017fs mutation (this study) was introduced [11]. Lentivirus packaging was conducted as described (http://cfm.brc.riken.jp/lentiviral-vectors/protocols/). Briefly, 17 μg of pCSII-EF-MCS-based plasmid containing *KIF11*^*WT*^ (± 3×HA tag), *KIF11*^*G268V*^ or *KIF11*^*S1017fs*^ (±3×HA tag) was co-transfected with 10 μg each of the packaging plasmid pCAG-HIVgp and the VSV-G/Rev-expressing plasmid pCMV-VSV-G-RSV-Rev into 293T cells using the calcium phosphate co-precipitation method. The medium was replaced after 16 h of transfection and the cells were cultured for a further 48 h. Viral particles were concentrated using Lenti-X-Concentrator (#631231, Takara Bio, Kusatsu, Japan) and the titer was measured with Lenti-X-GoStix (#631243, Takara Bio). U-2OS cells or HeLa cells were transduced at a MOI of 1 or 10, and stable clones were obtained by limiting dilution.

### Immunofluorescence

Eight to sixteen thousand cells were seeded on a poly-L-lysine-coated 18-mm square coverslip and cultured for 48 hours. The cells were then cultured in medium containing 10 μM MG132 (C2211, Sigma-Aldrich) with or without 4–8 μM STLC for 5 h followed by fixation with methanol at -20°C for 20 min. For some experiments, cells were pretreated with 9 μM RO-3306 for 15 h before addition of MG132 to accumulate cells into G2/M boundary. After fixation, the cells were blocked with PBS containing 2% BSA, and 0.1% Triton-X100 at room temperature for 30 min, treated with primary antibody for KIF11, HA or α-tubulin at 1:200 dilution overnight at 4°C, and subsequently with Alexa 488-conjugated goat anti-rabbit IgG antibody (A11008, Thermo Fisher Scientific), Alexa 488-conjugated goat anti-rat IgG antibody (A11006, Thermo Fisher Scientific), or Alexa 594-conjugated goat anti-mouse IgG antibody (A11005, Thermo Fisher Scientific) at 1:1000 dilution for 2 h. After washing, the coverslips were mounted with ProLong Gold Antifade Mountant (Thermo Fisher Scientific) and observed using a Nikon C1si confocal laser scanning microscope (Nikon, Tokyo, Japan). More than 100 metaphase spindles were counted for each condition and chi-square-test was performed to evaluate statistical significance of the difference in monopolar index. For some experiments, fluorescence intensity was quantified and analyzed using ImageJ/Fiji software [10].

## Results

### Isolation of STLC-resistant cells from two osteosarcoma cell lines

**Fig 1:**
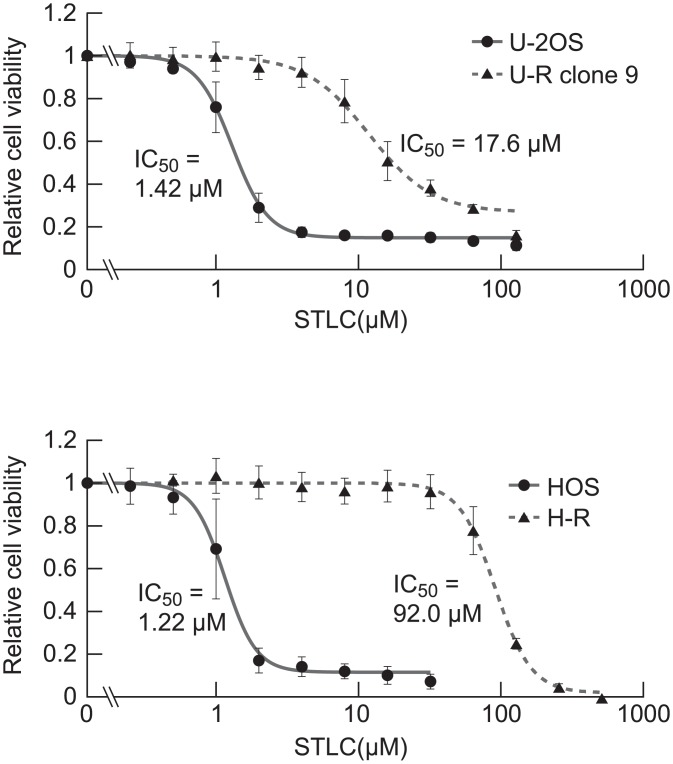
Isolation of STLC-resistant cells from two osteosarcoma cell lines. After chronic treatment of U-2OS and HOS cells with 2–8 μM STLC for 6 weeks, the cells were measured for STLC sensitivity. For the U-R cells derived from U-2OS, a single representative clone designated clone 9 was used. Relative cell viability for a given STLC concentration is shown as mean ± SD (n = 5).

Among six osteosarcoma cell lines we examined, chronic treatment of U-2OS and HOS with a sub-lethal dose of STLC successfully gave rise to STLC-resistant cells, which were designated U-R and H-R, respectively. For the U-R cells, one of several clones isolated from the surviving population, clone 9, was used as a representative. Their IC_50_s were increased to more than 10-fold relative to the parental cells (Fig 1, from 1.42 μM to 17.6 μM in U-2OS clone 9 and from 1.22 μM to 92.0 μM in HOS). Both U-R and H-R showed a moderate cross-resistance to another kinesin 5 inhibitor, monastrol (S1 Fig). Although the MG-63 line was able to acclimate to the medium containing 8 μM STLC, the IC_50_ of the survivors was only about 3-fold of that for the parental line (S2A Fig, 5.87 μM and 1.93 μM, respectively). The other three lines, G-292, NY and HuO9, barely survived the chronic treatment. All of the six lines expressed KIF11/kinesin-5 and KIF15/kinesin-12 to some degree (S2B Fig), while there was no clear correlation between their variation and survival after STLC treatment. The latter four cell lines were excluded from further analysis.

### Acquired STLC resistance is dependent on KIF15 but not on dynein on nuclear envelope

**Fig 2:**
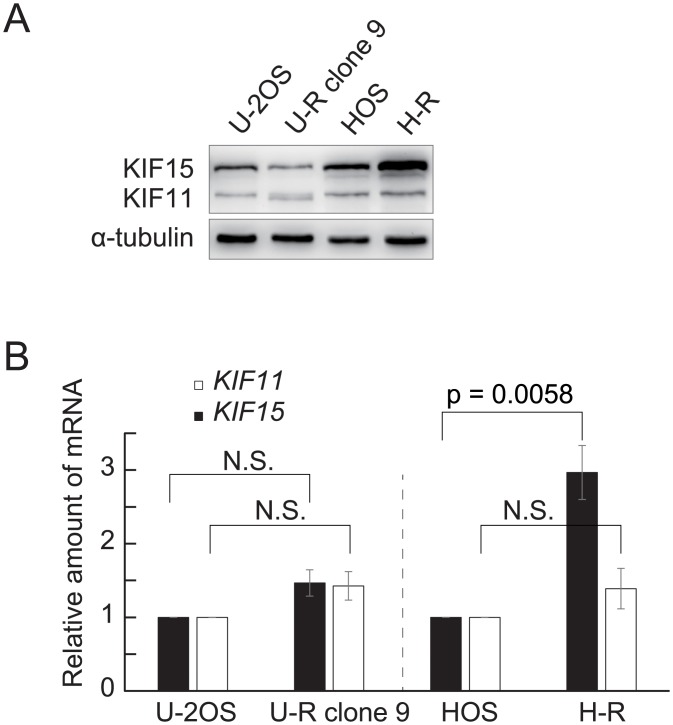
U-R cells express kinesin-5 with higher mobility. (A) The amount of KIF11 and KIF15 protein in the U-R and H-R cells was evaluated by immunoblotting. KIF15 was moderately increased in H-R cells relative to the parental HOS cells. Intriguingly, U-R cells expressed a KIF11 variant that migrated slightly faster on SDS-PAGE, in addition to normal KIF11. (B) mRNAs for *KIF11* and *KIF15* were quantified using qPCR and the results from five independent experiments were shown as mean ± SD. Two-tailed t-tests were used for statistical evaluation. *KIF15* mRNA in the H-R cells was significantly increased in comparison with the parental HOS cells, whereas no quantitative change in the U-R clone 9 was observed. For *KIF11* mRNA, neither the U-R cells nor the H-R cells showed any change in the expression level.

**Fig 3:**
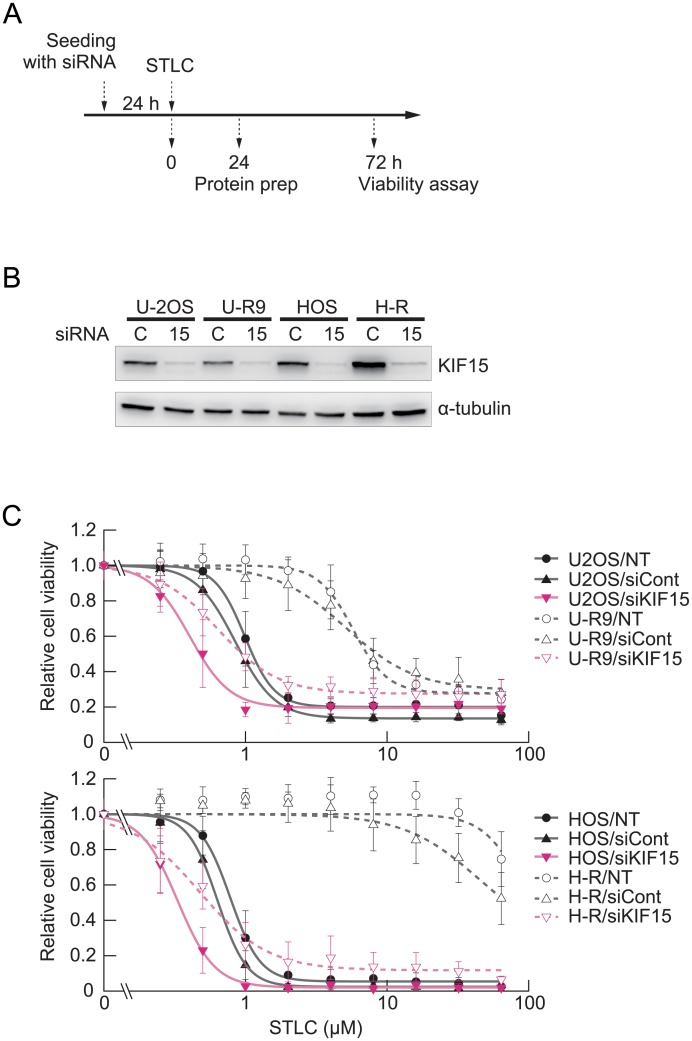
STLC resistance of both U-R and H-R cells is dependent on KIF15/kinesin-12. (A) Experimental procedure. (B) Evaluation of knockdown efficiency by immunoblotting. (C) The effect of KIF15 depletion on STLC resistance of U-R and H-R cells was examined by siRNA-mediated gene knockdown. Relative cell viability for a given STLC concentration is shown as mean ± SD (n = 3). KIF15 depletion substantially reduced the STLC resistance of U-R and H-R cells.

Given that previous studies had shown that cells overexpressing KIF15 were able to bypass the pharmacological inhibition of KIF11 [6,7], we first investigated whether the acquired resistance in the present case also involved a detectable change in KIF15 expression (Fig 2). Immunoblotting showed that the level of KIF15 protein was marginally decreased in U-R cells and was moderately increased in H-R cells relative to those in the parental lines (Fig 2A, 0.64-fold in U-R cells and 2.9-fold in H-R cells), whereas quantitative PCR of *KIF15* mRNA revealed a significant increase of the message only in H-R cells (Fig 2B). Nevertheless, STLC resistance appeared to be KIF15-dependent in both cases, as depletion of KIF15 substantially eliminated the STLC resistance of U-R and H-R cells (Fig 3, solid symbols). Interestingly, basal STLC resistance of the parental U-2OS cells and HOS cells also depends on KIF15 to some extent (Fig 3, open symbols). These observations agree with a notion that KIF15 expression is required for acquired and basal resistance to STLC in certain osteosarcoma cell lines. We next examined whether dynein on nuclear envelope is involved in the acquired resistance as previously suggested [6]. Depletion of dynein loader BICD2 modestly increased the STLC sensitivities of both the parental and the resistant cells (S3 Fig). The result suggests that dynein on nuclear envelope is not specifically associated with the acquired resistance of U-R and H-R cells.

### STLC-resistant cells derived from U-2OS express the C-terminal truncated kinesin-5 mutant

**Fig 4:**
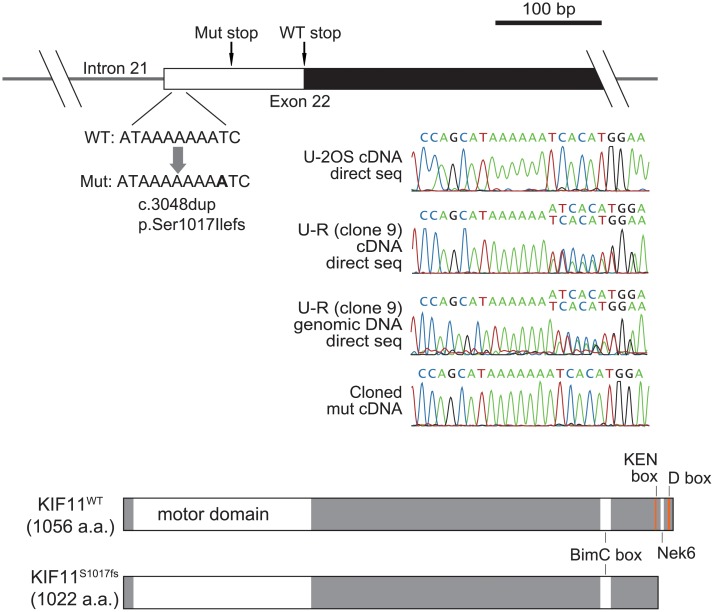
U-R cells harbor a frameshift mutation in one copy of the *KIF11* gene. Direct sequencing of *KIF11* cDNA identified a single A insertion in the A stretch of the last exon in U-R cells. This mutation would result in a frameshift accompanied by a 40-amino-acid truncation at the C-terminus containing a KEN-box and D-box. The relative height of the peaks in the electrophoretograms suggested that the mutant mRNA comprised about 35–40% of the total *KIF11* message.

Although there was no obvious quantitative change in the *KIF11* message between the parental lines and their STLC-resistant derivatives, immunoblotting revealed a KIF11 variant with slightly higher mobility in the U-R clone 9 cells (Fig 2A). No similar variant was seen in the H-R cells. Direct sequencing of *KIF11* cDNA as well as genomic DNA from the U-R clone 9 identified a single A insertion in the A stretch of the last exon (NM_004523.3:c.3048dup) that would result in a frameshift (S1017fs) and concomitant truncation of 40 amino acids at the C-terminus (Fig 4), thus agreeing with the immunoblotting result. This truncation eliminated a postulated KEN box (1,022–1,024 a.a.) and a D box (1,047–1,050 a.a.), while leaving the BimC box (917–936 a.a.) unaltered [12,13]. We isolated several independent clones from the U-R cells and confirmed that the immunoblot patterns and electrophoretograms for direct cDNA sequencing were identical among them (S4 Fig), indicating clonal expansion of the mutant throughout the U-R cell population, and that individual U-R cells expressed both the wild-type and the mutant KIF11 (KIF11^S1017fs^). Judging from the relative height of the peaks for cDNA between the wild-type and the mutant electrophoretograms, the mutant transcript comprised roughly 35–40% of the total *KIF11* message in the U-R cells. On the other hand, the amount of truncated KIF11 protein always appeared higher than that of the wild-type KIF11, probably due to lack of the sequences for protein degradation [12,13]. We were not able to find the identical *KIF11* mutation in COSMIC and ExAC databases.

### KIF15 obviates the essential function of KIF11 in H-R cells but not in U-R cells

**Fig 5:**
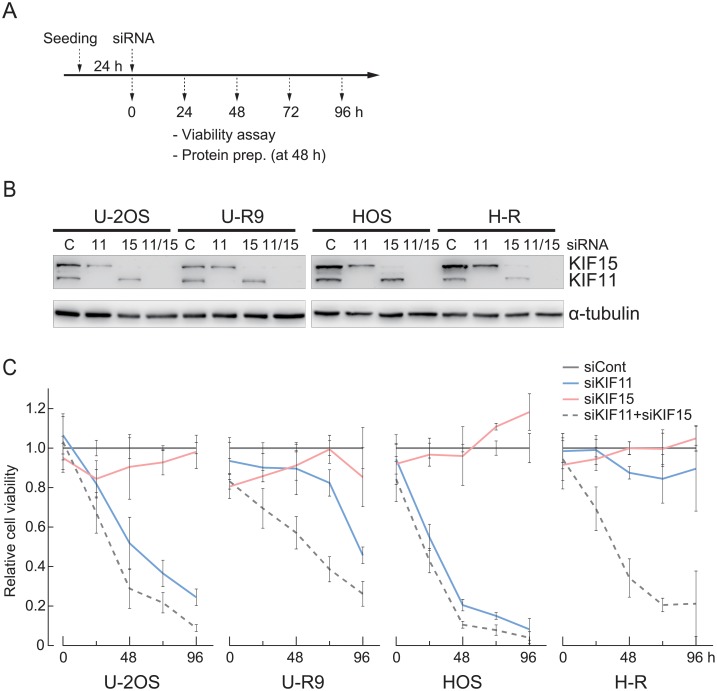
Effect of KIF11 or KIF15 depletion on growth of U-R or H-R cells. (A) Experimental procedure. (B) Evaluation of knockdown efficiency by immunoblotting. (C) *KIF11* knockdown heavily compromised the growth of the U-R cells, while the growth of H-R cells was affected only when both KIF11 and KIF15 were depleted, suggesting their mutually compensatory relationship. Cell viability was shown relative to that for the cells treated with control siRNA.

Previous studies had shown that cells with STLC resistance due to overexpression of KIF15 can undergo mitosis without KIF11 under STLC-free conditions [6,7]. Therefore, we investigated the effect of KIF11 or KIF15 depletion on the growth of U-R and H-R cells. At 48 h after *KIF11* siRNA transfection, the growth of U-2OS and HOS was markedly impaired compared with the cells treated with control siRNA, consistent with the need for KIF11 (Fig 5). The U-R clone 9 cells showed similar but slightly weaker dependency on KIF11. In contrast, growth of the H-R cells was apparently unaffected by KIF11 depletion alone and was noticeably impaired only when both KIF11 and KIF15 were depleted. This suggests that the two kinesins have a mutually compensatory or redundant relationship in H-R cells.

### Bipolar spindle formation in U-R and H-R cells is STLC-resistant

**Fig 6:**
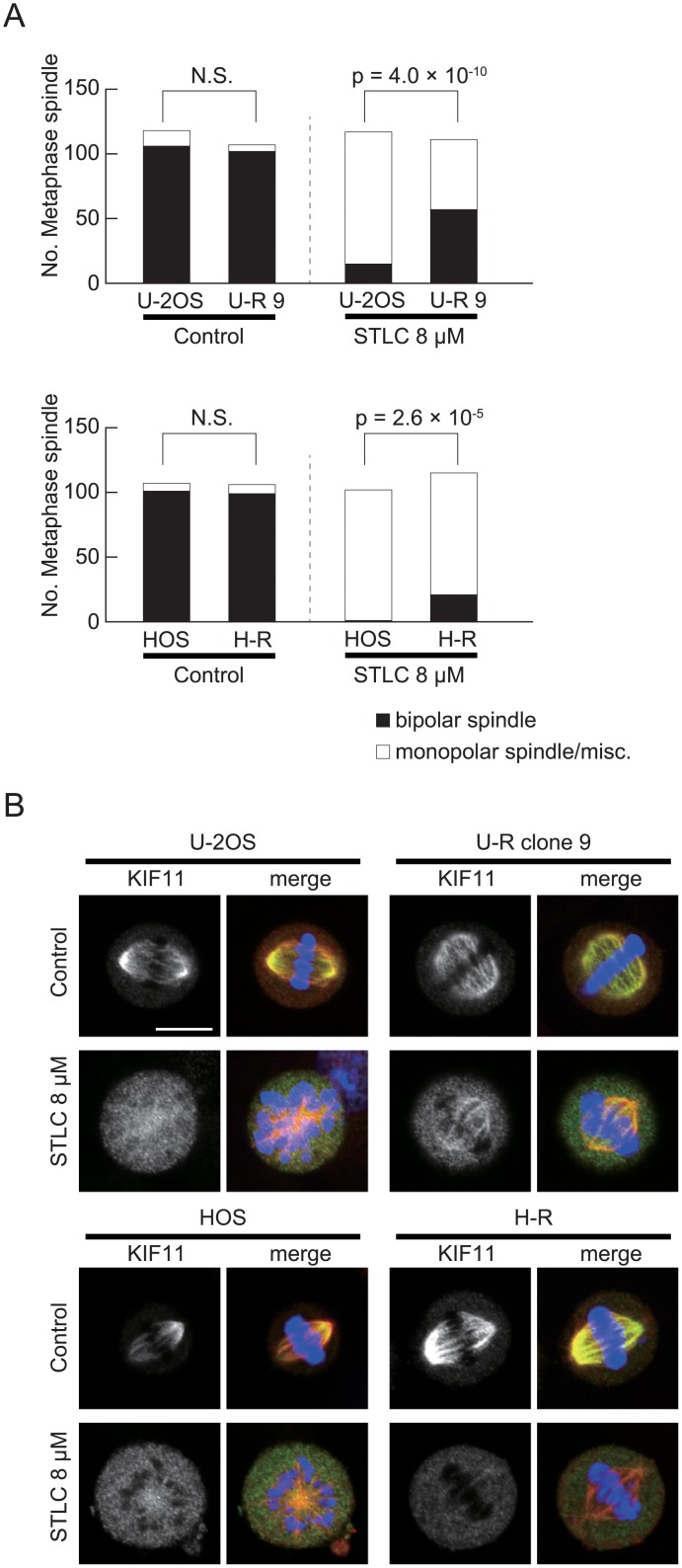
STLC-resistant bipolar spindle formation in U-R and H-R cells. (A) Cells were treated with 8 μM STLC for 5 h and spindle morphology was examined using immunofluorescence microscopy for α-tubulin. More than 100 metaphase spindles were counted for each condition and chi-square-test was performed to evaluate statistical significance of the difference in monopolar index. (B) Localization of KIF11 was examined using immunofluorescence microscopy. Some kinesin-5 was localized to the metaphase spindle in STLC-treated U-R cells, but was nearly absent in the spindle of STLC-treated H-R cells. Scale bar is 10 μm. Gray scale and green, kinesin-5; red, tubulin; blue, DNA.

We investigated how STLC treatment affects mitotic spindle formation in U-R and H-R cells by measuring the monopolar index (MPI). While the MPI of the parental U-2OS and HOS cells was 83% and 99%, respectively, after treatment with 8 μM STLC, the same treatment perturbed spindle bipolarity to a significantly lesser extent in the U-R clone 9 and H-R cells, which had MPIs of 53% and 82%, respectively (Fig 6A). We noticed that KIF11 in the U-R cells was preferentially localized to the bipolar spindles to some extent even in the presence of STLC (Fig 6B, relative fluorescence intensity at spindle region to non-spindle region is 1.60±0.166, n = 8), being a reminiscence of the rigor mutant KIF11G268V in HeLa cells [8]. In contrast, KIF11 was localized relatively uniform on the STLC-resistant bipolar spindles in the H-R cells (the relative fluorescence is 1.16±0.129, n = 7). Note that our STLC treatment condition is slightly milder than that in the previous report [8], which would explain residual KIF11 on the mitotic spindle even in the cells without KIF11 mutation.

### Ectopic expression of the C-terminal truncated mutant of kinesin-5-rendered U-2OS cells moderately resistant to STLC

**Fig 7:**
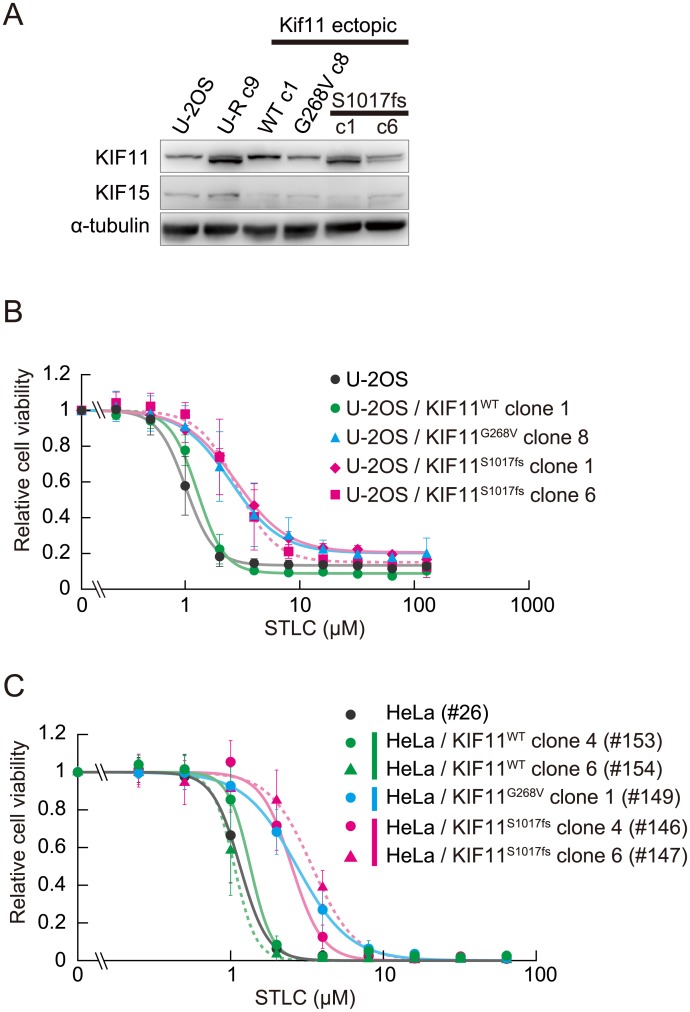
Ectopic expression of KIF11^S1017fs^ leads to moderate STLC resistance in U-2OS cells. (A) Immunoblot of the U-2OS cells expressing the *KIF11* transgenes. (B) STLC sensitivity of the U-2OS cells expressing the *KIF11* transgenes. Relative cell viability for a given STLC concentration is shown as mean ± SD (n = 5). Lentivirus-mediated transduction of *KIF11*^*S1017fs*^ rendered U-2OS moderately resistant to STLC, to the same degree as with the *KIF11*^*G268V*^ allele [8]. Overexpression of the wild-type allele failed to confer any notable resistance. (C) STLC sensitivity of the HeLa cells expressing the *KIF11* transgenes.

To show a causal relationship between the expression of the C-terminal truncated KIF11 mutant and STLC resistance, we ectopically expressed KIF11^WT^, KIF11^S1017fs^ or KIF11^G268V^ in U-2OS cells using lentivirus-mediated transduction and measured their sensitivity to STLC. Since we did not add an immunological tag to these transgenes in this set of experiments, expression of KIF11^S1017fs^ was evaluated from the relative intensities of signals with different sizes in the immunoblots, and that for KIF11^G268^ was evaluated from the relative heights of the peaks in electrophoretograms by direct sequencing of cDNA for *KIF11*. Protein expressed from the *KIF11*^*S1017fs*^ transgene showed a position in the immunoblot indistinguishable from that of the KIF11 variant in the U-R cells, confirming that the variant had originated from the frameshift mutation we identified (Fig 7). Two clones with different levels of KIF11^S1017fs^ expression (clones 1 and 6) showed a moderate STLC resistance (with IC_50_s of 3.69 μM for clone1 and 3.27 μM for clone 6, compared with 1.09 μM for U-2OS), to the same extent as a clone expressing KIF11^G268V^ (3.29 μM). In contrast, overexpression of the wild-type KIF11 failed to provide any marked resistance (1.38 μM). We stably expressed these *KIF11* alleles in HeLa cells to observe their effects on STLC survival in another context. Again, both KIF11^S1017fs^ and KIF11^G268V^ conferred moderate STLC resistance to the same extent, whereas overexpression of KIF11^WT^ did not lead to any notable resistance (Fig 7C).

### STLC-resistant localization of KIF11^S1017fs^ on mitotic spindle

**Fig 8:**
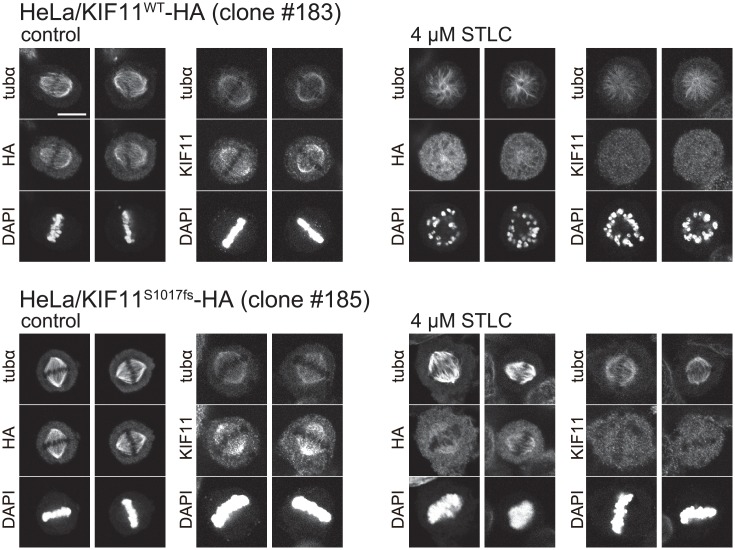
Localization of HA-tagged and bulk KIF11 on mitotic spindle. *KIF11*^*WT*^*-HA* or *KIF11*^*S1017fs*^*-HA* transfected HeLa cells were treated with 4 μM STLC and 10 μM MG132 for 5 h, and the HA-tagged and the bulk KIF11 localization was examined using immunofluorescence microscopy. Mitotic spindle assembly of the cells expressing KIF11^S1017fs^-HA showed a moderate STLC resistance, and KIF11^S1017fs^-HA showed a higher accumulation on bipolar spindle than bulk KIF11 in the presence of STLC. Scale bar is 10 μm.

To examine any possible difference in localization on mitotic spindle between the wildtype and the mutant KIF11, we introduced hemagglutinin (HA)-tagged version of the transgenes into HeLa cells and observed the bulk KIF11 and the tagged KIF11 in metaphase using immunofluorescence microscopy. In control metaphase, both bulk KIF11 and HA-tagged KIF11^WT^/KIF11^S1017fs^ showed a similar distribution on bipolar spindle, intensely accumulating on polar regions (Fig 8, left). When the cells were treated with 4 μM STLC, most mitotic spindles became monopolar in the cells expressing KIF11^WT^-HA (Fig 8, top right). In contrast, spindle assembly of the cells expressing KIF11^S1017fs^-HA showed a certain level of STLC-resistance (Fig 8, bottom right). Interestingly, while KIF11^S1017fs^-HA showed a moderate but clear STLC-resistant localization on bipolar spindle, the bulk KIF11 was almost uniformly distributed in the cell under this condition and localization on mitotic spindle was much weaker. Although we do not have a biochemical evidence, our observation may suggest that KIF11^S1017fs^ have a higher affinity to mitotic spindle than the wildtype KIF11 in the presence of STLC.

## Discussion

In the present study, we tried to identify a novel mechanism for acquired resistance to K5Is using six osteosarcoma cell lines. H-R cells, derived from the HOS cell line, conformed to a reported class of cells in which KIF15 almost completely substitutes for KIF11 when the latter is inhibited or depleted [6,7]. The substitution is considered to require ectopic localization of KIF15 to interpolar MTs, but how this occurs and whether overexpression of KIF15 alone is sufficient for its mislocalization is not clear. In some reported cases of KIF11-independent growth, the cells did not show a significant increase in the KIF15 level while their K5I resistance was still KIF15-dependent [6]. In these cells, quantitative or qualitative changes in certain proteins might assist the localization of KIF15 to interpolar MTs and coordinate MT-bundling. Dynein on the nuclear envelope in prophase is implicated in one case, but it is not currently known how universal it is and whether the dynein can functionally interact with KIF15 that has role mainly in prometaphase and metaphase. Although we do not have any evidences, it is possible that quantitative or qualitative changes in proteins other than KIF15 is also involved in the acquired STLC resistance of H-R cells. It may be noteworthy that KIF11 can sustain the growth of H-R cells when KIF15 is depleted. This suggests that two pathways for spindle assembly exist in a fully redundant manner and H-R cells preserve subcellular condition for KIF11 to function, even though it is now dispensable.

We have shown that a C-terminal truncation of KIF11/Eg5 contributes to the acquired resistance of U-R cells. The mutation responsible for the truncation was a single nucleotide expansion of an A stretch in the last exon of the *KIF11* gene, possibly resulting from genetic vulnerability of a certain mononucleotide runs [14,15]. Although previous studies have identified several KIF11 mutations mediating K5I resistance, virtually all of them were mapped to the N-terminal motor domain [8,16]. This is reasonable, since K5Is generally interfere with kinesin-5 function by targeting its N-terminal motor domain, and some of the mutations in this region are likely to render KIF11 refractory to K5Is. The KIF11^G268V^ rigor mutant constitutes a unique case in which bipolar spindle assembly is accomplished through cooperation of the mutant KIF11 with wild-type KIF15. The STLC resistance of U-R cells is similarly attributable to both KIF11^S1017fs^ and KIF15 as localization of KIF11^S1017fs^ to the mitotic spindle is STLC-resistant to some extent. These observations are consistent with the hypothesis that “division of labor” takes place in U-R cells, as in the case for KIF11^G268V^, although currently there is no further evidence for this.

How does C-terminal short truncation affect the functions of KIF11? Drosopoulos et al. have reported that the C-terminus of KIF11 harbors a KEN-box and a D-box, which are targeted by ubiquitin E3 ligase APC/CCdh1 in a cell-cycle dependent manner [13]. Quantitative regulation of KIF11 with these sequence motifs has been shown to be crucial for centrosome clustering in cells with supernumerary centrosomes, probably by limiting outward forces between centrosomes. It is noteworthy that the clustering-deficient phenotype due to C-terminally truncated KIF11 (1–951 aa) is antagonized by another K5I monastrol. The simplest explanation for the STLC resistance of U-R cells where both the KEN-box and D-box are similarly deleted (1–1016 aa) would be that the increased KIF11 antagonizes the inhibition of spindle assembly by STLC. However, this explanation does not accord with our observation that overexpression of wild-type KIF11 did not confer any notable resistance, as well as the fact that a difference in the expression level of KIF11S1017fs did not result in any marked difference in resistance. Furthermore, it does not account for the KIF15 dependency of STLC resistance. Nevertheless, it is still possible that—rather than the amount of KIF11 alone—fine cell-cycle-dependent tuning (in fact, loss of it) could be involved in STLC resistance. Other than the KEN-box and D-box, the truncated part of KIF11 contains the Nek6 phosphorylation site S1033, which is implicated in centrosome separation and/or spindle assembly [17, 18]. While we do not have any data to support a possible involvement of this phosphorylation in the acquired resistance to STLC, it seems rather counter-intuitive that loss of the phosphorylation critical for spindle assembly would enforce spindle assembly in the presence of K5Is.

C-terminal tail of KIF11 is also known to have a role in MT-bundling in unperturbed mitosis, since the low affinity of the N-terminal motor domain for MTs may not be sufficient for maintaining crosslinks between MTs in their homotetrameric configuration [19]. This short truncation might somehow enhance the affinity of the C-terminal tail for MTs, thus increasing their bundling activity and prompting KIF15 redistribution.

In contrast to U-2OS and HOS, the remaining four osteosarcoma cell lines did not develop STLC resistance despite their expression of KIF15. This difficulty in acquiring K5I resistance has not been reported previously, and may suggest that some unknown genetic or epigenetic conditions need to be fulfilled for resistance acquisition. p53 status clearly does not correlate with success or failure of K5I resistance induction, as p53 of U-2OS is wild-type and that of HOS is mutated according to COSMIC database. Clarification of these conditions may broaden our understanding of the general picture of the alternative pathway for spindle assembly.

At this moment, it is not very clear whether the low efficacy of K5Is in several clinical trials is in fact due to the acquired resistance through molecular alterations clarified by *in vitro* cell studies [5]. Nevertheless, we believe that information obtained from these studies would undoubtedly contribute to the development of the therapeutic application of K5Is.

## Supporting information

S1 Fig Monastrol sensitivities of U-2OS, HOS, and their STLC-resistant derivatives. (PDF)

S2 Fig (A) STLC sensitivity of MG-63 cells before and after chronic STLC treatment. Results from a single experiment are shown. (B) Expression of KIF15 and KIF11 in seven osteosarcoma cell lines. Saos-2 was not used in the present study, since its identity could not be verified by STR analysis.
(PDF)

S3 Fig (A) Experimental procedure. (B) Evaluation of knockdown efficiency by immunoblotting. (C) The effect of BICD2 depletion on STLC resistance of U-R and H-R cells was examined by siRNA-mediated gene knockdown. Relative cell viability for a given STLC concentration is shown as mean ± SD (n = 3).
(PDF)

S4 Fig Expression of a KIF11 variant with an increased mobility in U-2OS cells chronically treated with STLC as well as several clones isolated from them. n.c., not cloned.
(PDF)
