# Correction: Involvement of C-terminal truncation mutation of kinesin-5 in resistance to kinesin-5 inhibitor

**DOI:** 10.1371/journal.pone.0212821

**Published:** 2019-02-20

**Authors:** Eri Saeki, Shinji Yasuhira, Masahiko Shibazaki, Hiroshi Tada, Minoru Doita, Tomoyuki Masuda, Chihaya Maesawa

There is information missing from the Funding section. The complete, correct Funding information is as follows: H.T. and C.M. were funded by JSPS KAKENHI Grant Numbers JP16K20066 and JP17K08726 (https://www.jsps.go.jp/j-grantsinaid/). The funders had no role in study design, data collection and analysis, decision to publish, or preparation of the manuscript.

There is information missing from the Acknowledgements. The complete, correct Acknowledgments is as follows: We are grateful to Ms. Sachiko Yasui for technical assistance. This work was partly supported by JSPS KAKENHI Grant Numbers JP16K20066 to H.T. and JP17K08726 to C.M.
