# Supplementary figures and images for: Involvement of C-terminal truncation mutation of kinesin-5 in resistance to kinesin-5 inhibitor
Source: PLoS One. 2018 Dec 17;13(12):e0209296. doi: 10.1371/journal.pone.0209296 (PMC6296710; doi:10.1371/journal.pone.0209296)

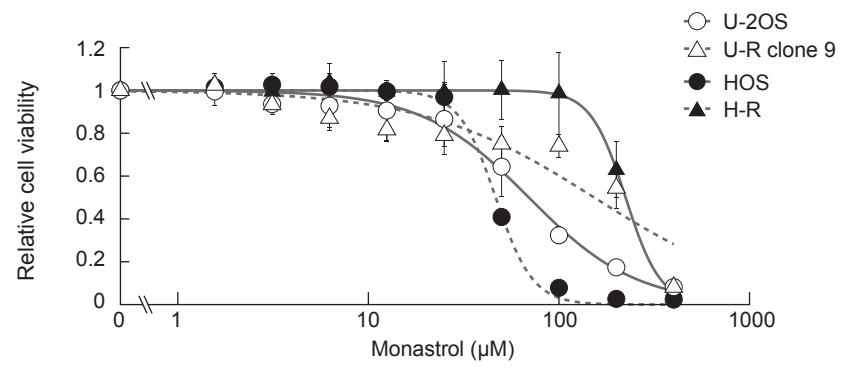

Supplement: S1 Fig — (PDF) [file pone.0209296.s001.pdf]

A

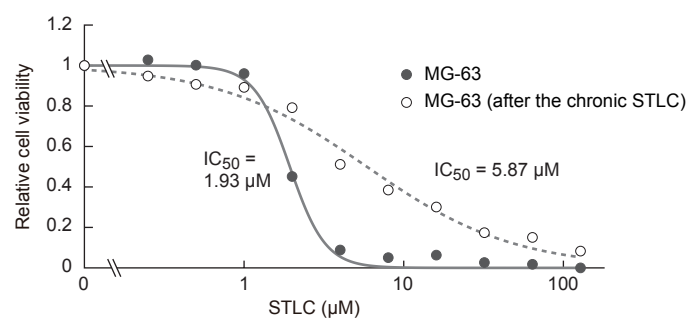

B

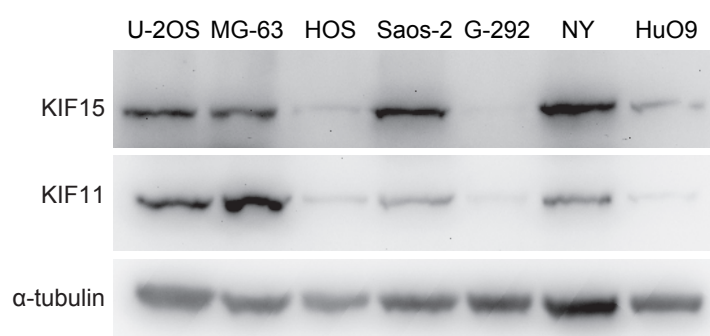

Supplement: S2 Fig — (A) STLC sensitivity of MG-63 cells before and after chronic STLC treatment. Results from a single experiment are shown. (B) Expression of KIF15 and KIF11 in seven osteosarcoma cell lines. Saos-2 was not used in the present study, since its identity could not be verified by STR analysis. (PDF) [file pone.0209296.s002.pdf]

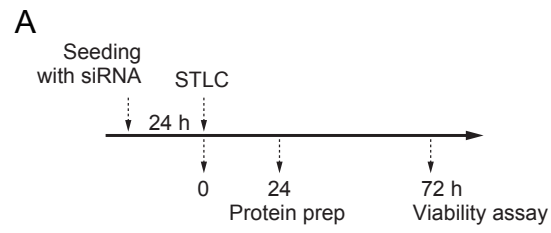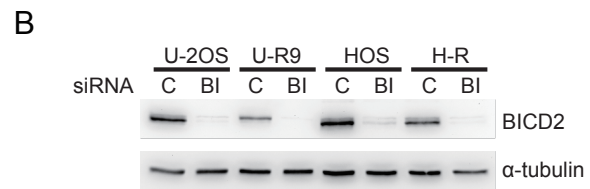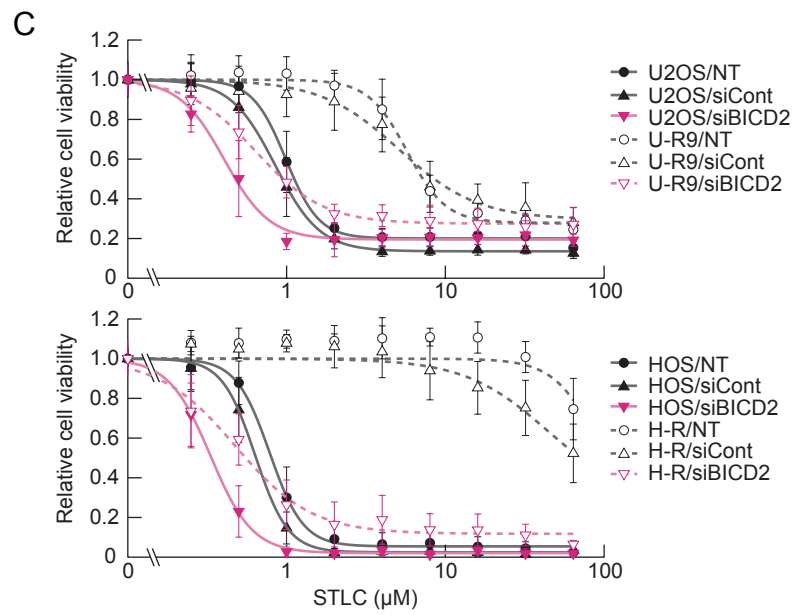

Supplement: S3 Fig — (A) Experimental procedure. (B) Evaluation of knockdown efficiency by immunoblotting. (C) The effect of BICD2 depletion on STLC resistance of U-R and H-R cells was examined by siRNA-mediated gene knockdown. Relative cell viability for a given STLC concentration is shown as mean ± SD (n = 3). (PDF) [file pone.0209296.s003.pdf]

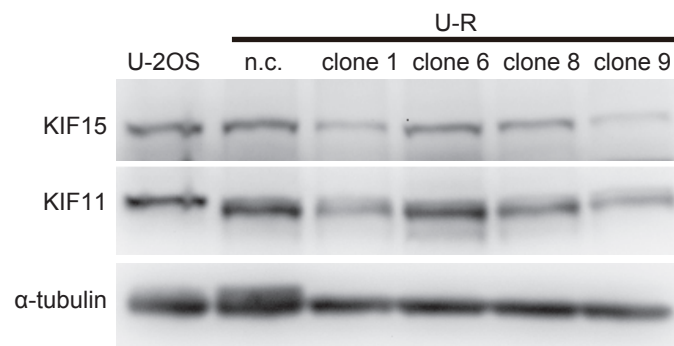

Supplement: S4 Fig — n.c., not cloned. (PDF) [file pone.0209296.s004.pdf]
